# Roles of microglia/macrophage and antibody in cell sheet transplantation in the central nervous system

**DOI:** 10.1186/s13287-022-03168-5

**Published:** 2022-09-11

**Authors:** Naoto Honda, Yasuhiro Watanabe, Yuta Tokuoka, Ritsuko Hanajima

**Affiliations:** Division of Neurology, Department of Brain and Neurosciences, Faculty of Medicine, Tottori University, 36-1 Nishi-cho, Yonago, Japan

**Keywords:** Cell sheet transplantation, Immune rejection, Mesenchymal stem cell, Microglia, Macrophage

## Abstract

**Background:**

We previously established a human mesenchymal stem cell (MSC) line that was modified to express trophic factors. Transplanting a cell sheet produced from this line in an amyotrophic lateral sclerosis mouse model showed a beneficial trend for mouse life spans. However, the sheet survived for less than 14 days, and numerous microglia and macrophages were observed within and adjacent to the sheet. Here, we examined the roles of microglia and macrophages as well as acquired antibodies in cell sheet transplantation.

**Methods:**

We observed the effects of several MSC lines on macrophages in vitro, that is, phenotype polarization (M1 or M2) and migration. We then investigated how phenotypic polarization affected MSC survival using antibody-dependent cellular cytotoxicity (ADCC) and phagocytosis (ADCP). We also confirmed the role of complement on cytotoxicity. Lastly, we selectively eliminated microglia and macrophages in vivo to determine whether these cells were cytoprotective to the donor sheet.

**Results:**

In vitro co-culture with MSCs induced M2 polarization in macrophages and facilitated their migration toward MSCs in vitro. There was no difference between M1 and M2 phenotypes on ADCC and ADCP. Cytotoxicity was observed even in the absence of complement. Eliminating microglia/macrophage populations in vivo resulted in increased survival of donor cells after transplantation.

**Conclusions:**

Acquired antibodies played a role in ADCC and ADCP. MSCs induced M2 polarization in macrophages and facilitated their migration toward MSCs in vitro. Despite these favorable characteristics of microglia and macrophages, deletion of these cells was advantageous for the survival of donor cells in vivo.

**Supplementary Information:**

The online version contains supplementary material available at 10.1186/s13287-022-03168-5.

## Background

Stem cell therapy provides considerable scope for the treatment of intractable diseases, including central nervous system (CNS) disorders. The brain has generally been considered immune-privileged [1]; however, recent advances in its understanding suggest that even the CNS is affected by various immune mechanisms, including innate and acquired immunity with both humoral and cellular aspects [2, 3]. Macrophages and microglia, the resident macrophage population in the CNS, are involved in both innate and acquired immunity. The “resting state” [4] microglia (M0), when activated, become polarized to express pro-inflammatory M1 and anti-inflammatory/tissue remodeling M2 phenotypes, as observed in macrophage activation [5, 6]. Harnessing immunity is key to the success of transplantation and regenerative therapy for CNS disorders.

Mesenchymal stem cells (MSCs) have emerged as potent transplantable reparative cells with unique properties, such as immunomodulation and trophic factor release [7]. Recent investigations have identified a regenerative propensity of MSCs to modulate macrophage phenotypes and functions via interleukin (IL)-4- or IL-13-mediated or microRNA-mediated pathways [8, 9]. We previously established a human MSC line named HAC-MSC that expresses hepatocyte growth factor, glial cell line-derived neurotrophic factor, and insulin-like growth factor 1 using a human artificial chromosome (HAC) [10]. We then confirmed that the transplantation of the cell sheet produced from this line resulted in longer survival of donor cells than when the cells were transplanted cell suspensions [11]. This procedure also contributed to the extension of the life span of mouse models of amyotrophic lateral sclerosis (ALS), a motor neuron disease [11]. During these experiments, we observed that numerous microglia/macrophage populations in the transplanted sheet area were of the arginase-1 (Arg1)-positive M2 phenotype, but some were of the nitric oxide synthase 2 (NOS2)-positive M1 phenotype. In the present study, we confirmed the effects of donor MSCs on the microglia/macrophage population in vitro. We also examined the effect of the microglia/macrophage population, acquired antibody, and the complement system on cultured MSCs. Last, we investigated how deletion of the microglia/macrophage population affected MSC sheet transplantation in vivo.

## Methods

### Cell culture and cell sheet

The HAC-MSCs [10] express reporter proteins such as green fluorescent protein (GFP), Emerald luciferase (Toyobo, Japan), and human neurotrophic factors. Human immortalized MSCs (hi-MSCs) [12] are maternal HAC-MSCs. KUM10 [13] is a naturally occurring immortal MSC mouse cell line. These MSCs were maintained in Dulbecco's modified Eagle's medium (DMEM, high glucose; D6429, Sigma-Aldrich, USA), 10% fetal bovine serum (FBS; FB-1365/500, Biosera, Fujifilm, Japan), and penicillin–streptomycin (PS, Fujifilm), and cultured at 37 °C with 5% CO_2_.

Cell sheets were prepared using an UpCell 48 multi-well plate (CellSeed, Japan) [11]. Each well (1.1 cm in diameter) was coated with FBS at 37 °C overnight, and the HAC-MSCs were seeded onto each well at 3 × 10^5^ cells/well and cultured overnight. The dish was incubated at room temperature (RT) for 30 min, and the HAC-MSCs were retrieved as a cell sheet.

### Mice

C57BL/6J mice (Shimizu Laboratory Supplies, Japan) were bred and maintained under specific pathogen-free conditions at the Tottori University Laboratory Animal Resources. All animal experimental procedures were approved by the Tottori University Animal Experiment Committee (approval number: h30-Y-024) to ensure the humane care and use of animals. The animals were euthanized by administering an overdose of pentobarbital anesthesia or CO_2_ treatment.

### Bone marrow-derived macrophage (BMDM)

The bone marrow in the femur and fibula was collected from 14- to 20-week-old C57BL/6J mice (*n* = 4–6), suspended in Cellbanker1 (Zenogen Pharma, Japan), and stored at − 80 °C [14]. The cells were thawed and cultured in RPMI1640 (Fujifilm) supplemented with 10% FBS, PS, and 20 ng/mL recombinant mouse macrophage colony-stimulating factor (BioLegend, USA) for 72 h. After washing with Dulbecco’s phosphate-buffered saline (Fujifilm), 0.25% trypsin/EDTA (Fujifilm) was added, and the BMDMs were collected. For M1 activation [15], interferon-γ (IFN-γ, BioLegend) and lipopolysaccharide (LPS; SC-3535, Santa Cruz, USA) were added to the medium at final concentrations of 10 ng/mL and 5 µg/mL, respectively. For M2 activation [15], IL-4 (BioLegend) was added to the medium at a final concentration of 20 ng/mL and incubated for 24–48 h.

### Flow cytometry (FCM)

BMDMs and MSCs were suspended in DMEM/FBS/PS and seeded onto a 24-well plate (1.5 × 10^5^ cells/mL) (Sumitomo Bakelite, Japan) and cultured for 48 h. The cells were collected in a suspension, blocked with anti-mouse FcR antibody (CD16/CD32, BD Bioscience, USA) in FCM buffer, containing Hanks' balanced salt solution (HBSS) (−) (Fujifilm) and 2% FBS for 20 min at 4 °C, and then stained with anti-mouse CD11b (clone: M1/70, PE-Cy7, BioLegend) as a pan-macrophage marker, anti-mouse CD80 PE/Cy5 (clone: 16-10A1, BioLegend) as an M1 marker, and anti-mouse CD86 APC (clone: GL-1, BioLegend) as an M1 marker for 20 min at 4 °C. Nuclear staining was performed with 4′,6-diamidino-2-phenylindole (DAPI; Dojindo, Japan) for 10 min at 4 °C. Cells were washed with FCM buffer, fixed in 4% paraformaldehyde (PFA) in phosphate-buffered saline (PBS) for 30 min at RT, and washed with PBS. Next, the cells were suspended in permeabilization buffer [0.5% saponin (Honeywell, USA) in PBS] for 15 min at RT and stained with anti-mouse CD206-PE antibody (Clone: C068C2, BioLegend) for M2 marker, or isotype control (Clone: RTK2758, Rat IgG2a κ, BioLegend) after washing with FCM buffer. The cells were then resuspended in FCM buffer and run on a BD LSRFortessa X-20 Flow Cytometer (BD Bioscience). Data were analyzed using the FlowJo software (Treestar, OR, USA). To identify the BMDM phenotype, CD11b was used for pan-macrophages, CD86 was used for the M1 phenotype, and CD206 was used for the M2 phenotype. DAPI was used to eliminate the dead cells. Living cells were stained with CytoRed (Dojindo).

### Transwell migration assay

Transwell assays were performed according to the manufacturer’s protocol to determine whether MSCs could enhance the migration of BMDMs. Each MSC line was suspended in DMEM/FBS/PS on a 24-well plate (1.5 × 10^5^ cells/500 µL) and incubated for 4 h until the cells adhered to the bottom surface. Pore transwells (8.0 µm, Corning, USA) were set in the wells and seeded in upper layers with BMDM (1.5 × 10^5^ cells). Following incubation for 24 h, the number of migrating cells was counted. The transwells were collected and fixed with 4% PFA for 20 min and washed with PBS, and the upper surface of the membrane was cleared. The membrane was cut and attached to a glass slide and mounted with Vectashield containing DAPI (Vector Laboratories, USA). Images of the entire membrane were captured with a BZ-9000 fluorescence microscope (Keyence, Japan), and the images were merged with a BZ-II Analyzer (Keyence). The combined images were used to measure the number of cells using the ImageJ software [16].

### Antibody-dependent cellular cytotoxicity (ADCC) and antibody-dependent cellular phagocytosis (ADCP)

We next investigated ADCC in MSCs [17]. MSCs (1.5 × 10^5^ cells) were injected intraperitoneally to 14-week-old C57BL/6 mice to facilitate the production of specific antibodies against MSCs. Two weeks later, blood was collected from the hearts of two mice, incubated at 4 °C overnight, and centrifuged for 5 min at 6000*g* to collect the serum. The serum obtained using this procedure was termed “sensitized serum” and that collected without following the aforementioned processes was termed “normal serum.” Sera with and without heat inactivation of the complement were prepared to see the role of the complement system. BMDMs (1.5 × 10^5^/well) were seeded in 24-well plates and induced to M1 and M2 polarization for 24 h. BMDMs cultured without induction were defined as M0 and empty plates without BMDMs were termed BMDM (−). After the induction period, all wells were washed twice with DMEM/FBS (−), following which the HAC-MSCs were added at 1.5 × 10^5^ cells/well, and cultured with DMEM containing 10% sensitized or normal serum for 24 h. Collected cells including the floating ones were analyzed using FCM.

ADCC was determined by DAPI-positive (dead) cells in the CD11b-negative fraction. DAPI cannot penetrate unfixed (viable) cells to reach the nucleus but can penetrate cell membranes of dead or dying cells (Additional file 1: Fig. S1). BMDMs were identified as the CD11b-positive population, and MSCs were GFP-positive and identified as the CD11b-negative population. ADCC was expressed as a percentage of DAPI-positive cells among the total CD11b (−) cells.

ADCP [18] was expressed as a percentage of GFP-positive populations within the CD11b-positive (BMDM) fractions. We considered a GFP-positive BMDM population, wherein the BMDMs processed HAC-MSCs that expressed GFP.

### Clodronate-encapsulated liposome (CLP) treatment and transplantation

Phagocytic cells recognize CLPs as foreign particles and proceed to remove them via phagocytosis. The liposomes then release clodronate into the cytosol, resulting in cell death. Non-encapsulated clodronate cannot cross the cell membrane to initiate cell death. Thus, similar to colony-stimulating factor 1 receptor inhibitors such as PLX5622 [19], CLPs can deplete the microglia/macrophage population. CLPs (10 mg/mL; Hygieia Bioscience, Japan) were administered by intrathecal injection (5 µL) 3 days before transplantation to selectively eliminate host microglia and macrophages [20] (Additional file 1: Fig. S2). Additionally, CLPs were administered by intraperitoneal injection (25 µL) immediately prior to transplantation. As a control, liposomes were administered.

Transplantation was performed according to a previously described procedure [11]. In brief, after exposing the mouse skull, a rectangular area of the left cerebral cortex approximately 5 mm (width) by 4 mm (length) was surgically exposed, and a cell sheet was transplanted. For the control group, sham surgery was performed by exposing the brain surface without the application of a cell sheet. We did not use immunosuppressive agents during the operation period.

### In vivo imaging

For in vivo observation, HAC-MSCs were quantified by in vivo luciferase imaging using the Xenogen IVIS Lumina system (Summit Pharmaceuticals International Corporation, Japan) from 0 to 14 days. After transplantation, the cell sheets were transplanted into mice and the emission signals were monitored at five time points: immediately after transplantation, 1, 3, 7, and 14 days post-operation (exposure time of 1 min). The mice were anesthetized with 2% isoflurane (AbbVie, Japan) and 100% O_2_. Intraperitoneal injection of d-luciferin (150 mg/kg, Cayman Chemical, USA) was performed 30 min before observation. The bioluminescence emitted from the HAC-MSCs was analyzed using IVIS Live image 2.60 software (Summit Pharmaceuticals, Japan). The region of interest was set around the transplanted area. Changes in luminescence were calculated using the value at day 0 as a standard for each mouse. ​

### Immunohistochemistry

Animals were deeply anesthetized with pentobarbital (Kyoritsu Pharma, Japan) and transcardially perfused with saline followed by 4% PFA. The brains were removed, fixed in 4% PFA for 18 h, and immersed in 30% sucrose in PBS at 4 °C for 3–7 days. Frozen sections of 30 µm thickness were prepared using a microtome (Leica, Germany). Sections were blocked with 5% bovine serum albumin (Sigma-Aldrich) or 5% donkey serum (Fujifilm) in PBS for 1 h at RT, and frozen sections were stained with goat anti-Iba1 IgG (Fujifilm), rabbit anti-arg1 IgG (GeneTex, USA), rabbit anti-NOS2 IgG (Bioss, USA), donkey anti-rabbit IgG-Alexa flour 555 (Abcam, UK), and donkey anti-goat-IgG-Alexa flour 405 (Abcam). The primary antibody reaction was performed overnight at 4 °C. Sections were incubated with secondary antibody at 4 °C for 3 h and then mounted with Vectashield (Vector laboratories) containing DAPI. Stained sections were observed using a BZ-9000 fluorescence microscope (Keyence) or an LSM780 confocal microscope (Carl Zeiss, Germany).

### Statistical analysis

For statistical analysis, the Student’s *t* test was used for the comparison of two groups and one-way analysis of variance (ANOVA) was performed for multiple comparisons. For post analysis, Shaffer's modified sequentially rejective Bonferroni procedure was employed using the R software (version 4.1.2) [21]. Statistical significance was set at* p* < 0.05.

## Results

### Phenotype of infiltrating microglia

**Fig. 1:**
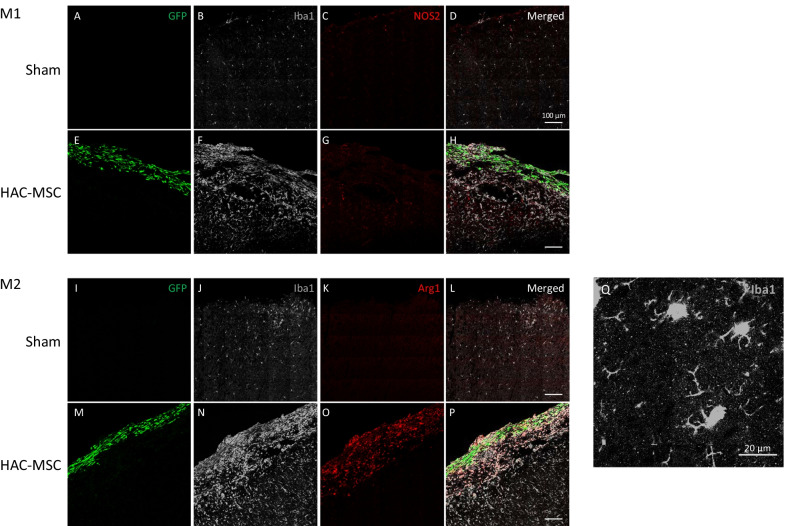
Infiltrating microglia and macrophages were dominantly M2 phenotype. HAC-MCS-transplanted (expressing GFP) or sham-operated brain tissues were stained with microglia marker (Iba1, gray), M1 marker (NOS2, red), or M2 marker (Arg1, red), and observed by confocal microscopy

Frozen sections were obtained from sham-operated or HAC-MSC-transplanted mice 7 days after transplantation and stained with antibodies against pan-microglia/macrophage (Iba1, gray), M1 (NOS2, red), or M2 (Arg1, red) markers. HAC-MSCs intrinsically express GFP (Fig. 1A, E, I, M). In the sham-operated group, some mice showed mild microglial activation at the exposed brain surface, but only a few cells were positive for M1 or M2 markers (Fig. 1B–D, J–L).

More Iba1-positive cells were found in the HAC-MSC transplantation group than in the sham group (Fig. 1B, F, J, N). These Iba1-positive cells primarily expressed the marker for M2 (Fig. 1N, O), while a small number of them also expressed the M1 marker (Fig. 1F, G). The M2-polarized cells were particularly abundant within and beneath the HAC-MSC sheets (Fig. 1M–P). Accompanied by phenotypic polarization to M1 or M2 phenotype, infiltrating microglia/macrophage populations had three features: great numbers, enlarged cell bodies, and fewer branches, which were judged as the activated phenotype (Fig. 1Q).

## MSCs facilitate M2 polarization of BMDMs in vitro

**Fig. 2:**
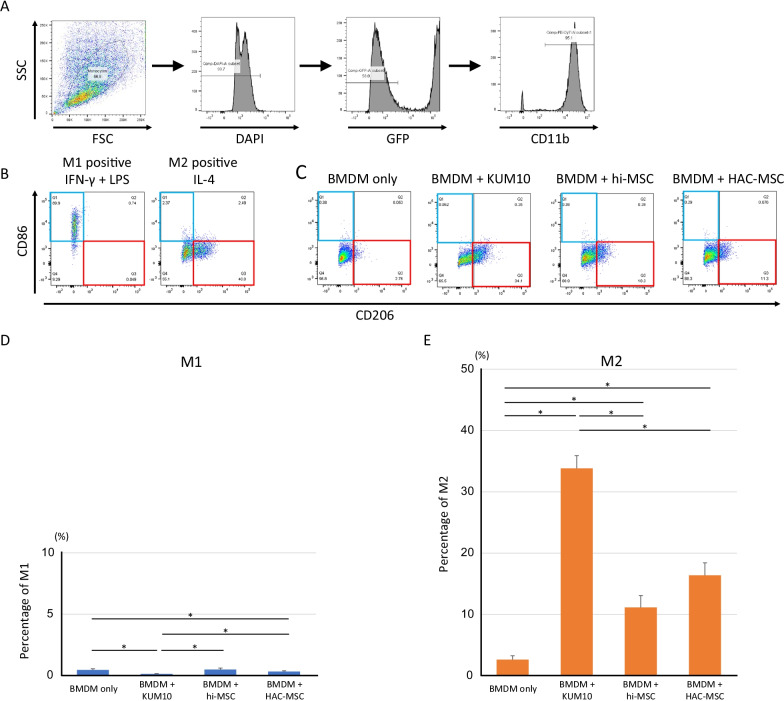
MSC facilitated the polarization of BMDM to M2 phenotype by co-culture. BMDM population was fractionated by forward scatter (FSC) versus side scatter (SSC) analysis. The expression of CD86 (M1 marker) and CD206 (M2 marker) in the BMDMs (DAPI-GFP-CD11^+^) fraction was detected by FCM (**A**). The positive control for M1 was induced by the stimulation of IFN-γ and LPS (blue square), and for M2, positive cells were produced by the stimulation of IL-4 (red square) (**B**). BMDMs co-cultured with KUM10, hi-MSC, HAC-MSC, or BMDM alone were cultured for 48 h (**C**). M1 and M2 percentages in each condition (*n* = 12) are expressed as the mean ± standard error (S.E.) (**D**, **E**). One-way ANOVA was performed within each phenotype with post hoc analysis: Shaffer's modified sequentially rejective Bonferroni procedure. *p < 0.05

FCM was used to detect the CD86 (M1 marker) and CD206 (M2 marker) expression of the BMDM fraction (DAPI, GFP, and CD11b-positive) in the side scatter (SSC) versus the forward scatter (FSC; Fig. 2A). M1-positive controls were obtained from BMDMs stimulated by IFN-γ and LPS, while M2-positive controls were obtained by those stimulated by IL-4 [22] (Fig. 2B). BMDMs were either cultured alone or co-cultured with KUM10, hi-MSCs, and HAC-MSCs for 48 h, and M1 and M2 activation markers of BMDMs were detected using FCM (Fig. 2C). The percentage of M1 or M2 in the CD11b-positive fraction is shown in Fig. 2D and E. Although statistical differences were observed, there was very little M1 activation (< 1%) compared to that observed in the M1-positive control in all co-culture conditions (Fig. 2D). In contrast, the percentage of cells activated to the M2 phenotype was significantly higher in co-culture with any MSCs than that in BMDM alone (> 30% in co-culture with KUM10, > 10% with hi-MSCs, and > 15% with HAC-MSCs; Fig. 2E). This finding may be because KUM10 was originally derived from the bone marrow of the same mouse strain as BMDM, and therefore, its M2 polarizing effect was stronger than that of human-derived MSCs. There was no statistically significant difference in the percentage of M2 activation between hi-MSCs and HAC-MSCs.

## MSCs enhance BMDM migration

**Fig. 3:**
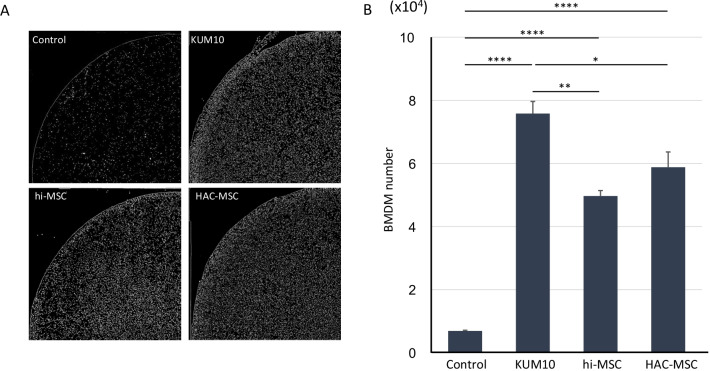
MSC facilitated the migrating ability of BMDMs without direct contact. BMDMs were cultured in the upper layer and lower layer of MSCs for 24 h. Migrated BMDMs were stained with DAPI (Gray) (**A**) and counted. All MCS types enhanced the migration of BMDM; KUM10 facilitated the migration most strongly (**B**). Data are expressed as the mean ± S.E. (*n* = 4). *p < 0.05, **p < 0.01, ****p < 0.0001

To confirm the migration ability of BMDMs in the presence of MSCs, a transwell assay was performed. We seeded 1.5 × 10^5^ of each type of MSC in 24 multi-wells and seeded 1.5 × 10^5^ BMDMs on the top surface of the transwell, which were then incubated for 24 h. The BMDMs remaining on the top surface were completely removed, and the nuclei of BMDMs migrating to the bottom were stained with DAPI (Fig. 3A). The acquired images were analyzed using ImageJ Fiji to measure the number of BMDMs (Fig. 3B). Out of the 1.5 × 10^5^ BMDMs, approximately 1.0 × 10^4^ cells migrated when there were no MSCs in the lower wells. In the presence of HAC-MSCs, approximately 6.0 × 10^4^ BMDMs migrated, which was not significantly different from the number of migrated cells in the presence of hi-MSCs (6 × 10^4^ cells). KUM10 significantly increased the migration ability of BMDMs compared to any other MSCs.

### ADCC and ADCP activities

**Fig. 4:**
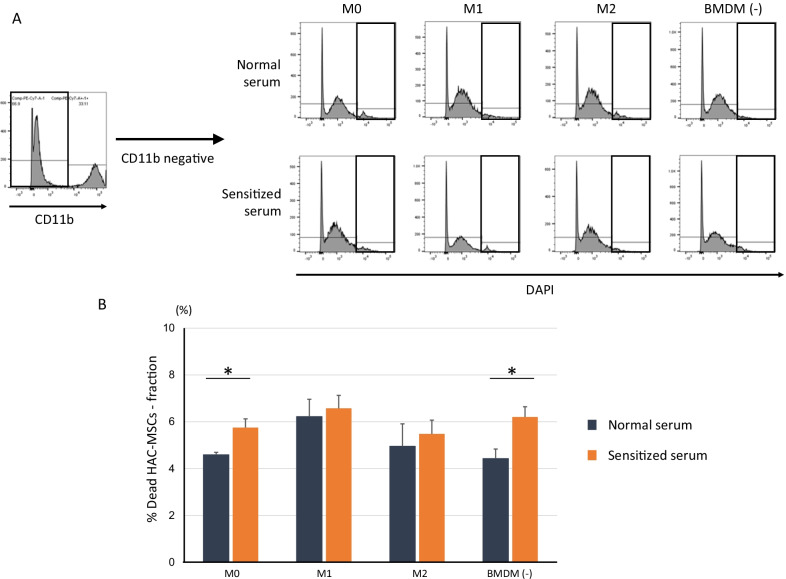
HAC-MSC-sensitized serum enhanced ADCC. DAPI-positive dead cells in the CD11b-negative fraction were collected after 24 h of co-culture of BMDMs and HAC-MSCs. M0: BMDM (medium only), M1: BMDM (LPS + IFN-γ stimulation 24 h), M2: BMDM (IL-4 stimulation 24 h), BMDM (−): no BMDM. Non-BMDM dead cell state for each BMDM-activated phenotype is shown (**A**). The *t* test between normal and sensitized sera within the corresponding BMDM activation phenotypes (**B**). Differences between normal and sensitized sera in M0 and BMDM (−) groups were observed. Data are expressed as the mean ± S.E. (*n* = 4, each phenotype). There were no significant differences among the sensitized groups (M0, M1, and M2) or normal groups

ADCC activity in M0 BMDMs significantly increased when cultured with sensitized serum compared to normal serum. Under normal serum conditions, M1-polarized BMDMs showed higher ADCC activity than M0 (Fig. 4A, B). Surprisingly, without co-culture with BMDM, the cytotoxic activity was significantly increased in sensitized serum compared to normal serum, indicating that cytotoxicity partly occurred in a BMDM-independent manner (Fig. 4B).

**Fig. 5:**
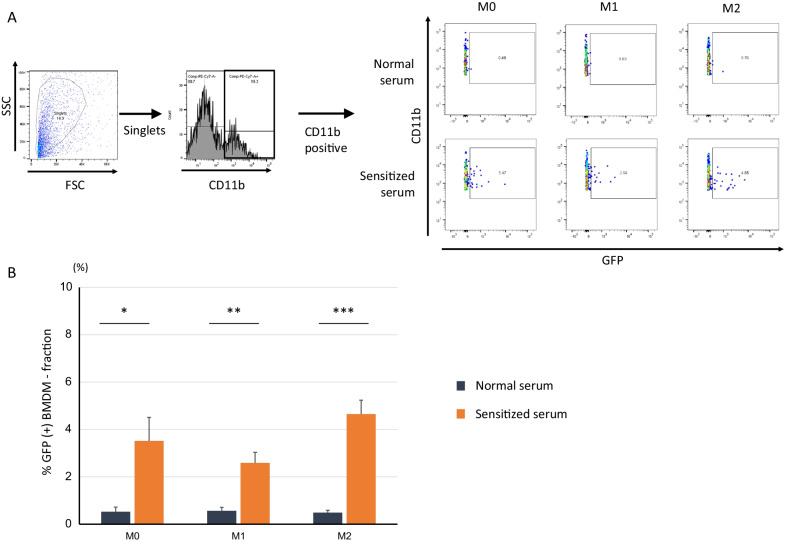
ADCP in BMDMs was enhanced by sensitized serum. Representative FCM results are shown in (**A**). M0: Medium only; M1: LPS + IFN-γ stimulation 2, M2: IL-4 stimulation. **A** GFP-positive cells in singlets (FSC vs. SSC) and CD11b-positive fraction of cells collected after 24 h of co-culture. The percentage of GFP-positive cells in the CD11b-positive fraction is shown (**B**). The *t* test between normal and sensitized sera within the corresponding BMDM activation phenotypes. Data are expressed as the mean ± S.E. (*n* = 4, each phenotype). There were significant differences between normal and sensitized sera in M0, M1 and M2 phenotypes. There were no differences among the sensitized groups (M0, M1, and M2) or normal groups. *p < 0.05, **p < 0.01, ***p < 0.001

As for ADCP (Fig. 5), following treatment with or without polarization, BMDMs were co-cultured with HAC-MSCs in the presence of normal or sensitized serum. After 24 h, BMDM-phagocytosed HAC-MSCs were identified by GFP fluorescence, as GFP was only expressed in HAC-MSCs (Fig. 5A). With normal serum culturing, ADCP was seen in less than 1% of the population in any of the co-cultures with M0, M1, or M2 phenotypes (Fig. 5B). In contrast, under sensitized serum conditions, an increase of 3.5%, 2.5%, and 4.5% in M0, M1, and M2, respectively, was confirmed compared to normal serum conditions (Fig. 5B).

### Complement inactivation on cytotoxicity

We further compared the sera with and without heat inactivation on ADCC activity in the presence of BMDMs (M0) and their absence: BMDM (−). Deactivation of complement showed no significant effect on survival of MSCs in co-culture with BMDM (M0; Additional file 1: Fig. S3A) and in BMDM (−) (Additional file 1: Fig. S3B), indicating that the toxicity is complement-independent [23].

### Cell sheet transplantation with or without CLP treatment

**Fig. 6:**
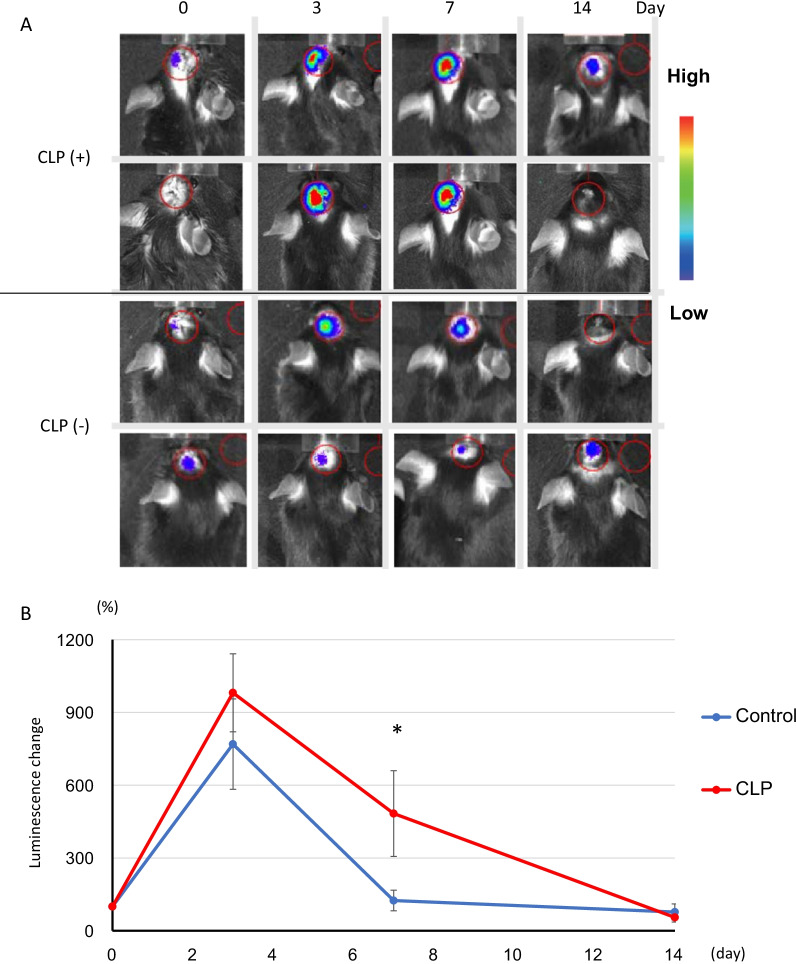
Deletion of microglia/macrophage population intensified the survival of HAC-MSC sheet. The luminescence level on day 0 was set to 100%, and the trend of luminescence level was calculated for CLP-treated [CLP (+)] and empty liposome [CLP (−)] groups (**A**). A significant difference between CLP (+) (red) and CLP (−) (blue) groups was identified on day 7 after transplantation (*n* = 10, each group) (**B**). Data are expressed as mean ± S.E. A *t* test was performed for comparison between groups on each day

Cell sheet transplantation results (Fig. 6A) indicated that the average luminescence was tenfold higher in the CLP-treated group on day 3 (compared with day 0 as 100%) and then decreased after day 3. On day 7 following transplantation, the CLP group showed an average fivefold increase in luminescence compared to day 0, although the luminescence was lower than that on day 3, indicating a significant increase in cell-sheet-derived signal intensity in the CLP group. In contrast, on day 14, the signal decreased immediately after transplantation, and the luminescence on day 7 decreased to almost the same level as that on day 0 in the control group. Additionally, on day 14, the signal was lower than that on day 0 (Fig. 6B).

## Discussion

### Immune rejection of donor tissue

There are multiple physiological and immunological barriers against the grafting of donor tissue [2]. Even syngeneic transplantation, donor cells, or cell sheets have been reported to disappear rapidly from the host, for example, after cell injection in a mouse model of hindlimb ischemia [24] and cell sheet transplantation in the heart of rats with myocardial infarction [25]. First, because of the absence of existing blood vessels in cellular grafts, the cell graft results in hypoxic and low-nutrient conditions a few hours post-transplantation. According to a report [2], environmental changes, including hypoxia, rather than subsequent immune responses, are the main causes of the low survival rate (typically < 30% of grafted cells) associated with cell transplantation in the brain. Second, natural antibodies against donor cells may be followed by complement activation. Natural antibodies also cause infiltration of innate immune cells (such as natural killer cells and neutrophils) into grafts [2]. Macrophages and microglia also begin to infiltrate during this stage. During these early phase reactions, the presence of macrophages is reported to harm rather than benefitting the donor cells since macrophage depletion reduces the activation of CD4^+^ T cells, and causes the depletion of tumor necrosis factor-α, interleukin-1, and nitric oxide [2]. Third, cellular rejection occurs within days to weeks after transplantation, and may involve T cells, innate immune cells, or both [2]. In this stage, specific antibodies against donor cells start to increase, further intensifying the cytotoxicity against donor cells; roles of microglia/macrophage populations (and complement system) could be significant at this stage.

### Cell sheet transplantation to the CNS

To date, there are two reports [26, 27] that describe cell sheet transplantation in the CNS, both are related to ischemic stroke in rat models. Seven days following permanent middle cerebral artery occlusion (MCAO), rats were transplanted with rat bone marrow stromal cell (BMSC) sheets onto the ipsilateral neocortex [26]. Four weeks post-transplantation, the donor BMSCs were still distributed to the neocortex adjacent to the cerebral infarct and expressed a neuronal phenotype. Cell sheet transplantation significantly improved motor function in the animals compared to that in vehicle-injected animals. The BMSC sheet did not induce reactive astrocytes in the adjacent neocortex; however, the presence or absence of activated microglia and macrophages was not mentioned in this study. In another report [27], 2 days following the MCAO procedure, rats were transplanted with a rat adipose-tissue-derived MSC sheet. Approximately 14 days after transplantation, significant angiogenesis and neurogenesis were observed, accompanied by behavioral improvement. Transplanted cells were identified within newly formed perivascular walls as pericytes. Moreover, newly formed blood vessels within the cell sheet were anastomosed to the cerebral blood vessels in the host. However, this study also failed to determine the status of microglia and macrophages.

Thus, since roles of microglia/macrophage populations in shell sheet transplantation in the CNS have been paid relatively little attention, successful translation needs more and more understanding of each player in immune rejection, including microglia and macrophages.

### Acute and delayed rejection in our experiments

In the present study, in vitro investigation revealed that both mouse-derived and human-derived MSCs facilitated the M2 polarization of BMDMs, and the presence of MSCs significantly promoted their migration. There were no significant differences in ADCC and ADCP activities among the M0, M1, and M2 phenotypes. Cytotoxicity could occur through humoral factors alone, and, similar to ADCC, sensitized serum enhanced toxicity. These results could suggest that even in vivo transplanted MSCs not only exert their cytoprotective effects on the host but also change the host environment. Despite the aforementioned effects on microglia and macrophages, in vivo observations revealed that the depletion of the microglia/macrophage populations during transplantation intensified the survival of the MSC sheet on day 7 following transplantation, which indicated that the presence of the microglia and macrophages was more harmful than beneficial in this transplantation procedure. Up to 14 days after the transplantation, however, regardless of the absence or presence of microglia/macrophage population, no surviving donor sheet cells were found. We speculate that this timing coincides with the upsurge of specific antibodies against donor MSCs that usually appear within a couple of weeks after sensitization (or immunization). Throughout this period, ADCC continues to present with the involvement of the microglia/macrophage population.

Our findings shed light on the importance of microglia and macrophages in cell sheet transplantation in the CNS. In addition, our report indicated that circulating antibodies (innate or acquired) play a role in immune rejection of donor cells. Recent advances in medicine could offer promising methods for dealing with various types of immune rejection. Practically, immunosuppressive agents such as FK506 or cyclosporin are used in medical practice against late-phase (acquired) immune rejection. Just as CLP can be used to deplete microglia and macrophage populations, it is also possible to deplete immunoglobulin-producing plasmacytes. For example, inebilizumab, a monoclonal antibody (mAb), binds to and depletes CD19^+^ B cells, including plasmablasts and plasmacytes [28], leading to immunoglobulin depletion.

## Conclusions

In this study, we used BMDMs in in vitro experiments, while we did not demarcate macrophages from microglia in the in vivo transplantation experiments.

The present study concluded that deletion of microglia and macrophage populations is better for graft survival. Although our data failed to indicate cytotoxic effects between M1 and M2 phenotypes regarding ADCC, better graft survival may be achieved by selectively suppressing the M1 population, intensifying the M2 population, or both.

## Supplementary Information

**Additional file 1: Fig. S1.** Morphological analysis of DAPI-positive cells in DCM. After FCM, HAC-MSC fraction (CD11b-negative and DAPI-positive cells) were observed. Living cells were stained with CytoRed. CytoRed-negative and DAPI-positive cells were observed (arrows). **Fig. S2.** CLP administration depletes microglia and macrophage population. Confocal microscopy images of empty liposomes injected (**A**–**C**) or CLP-treated (**D**–**F**) brain surface adjacent to the HAC-MSC sheet. GFP, green; Iba1, gray. **Fig. S3.** Complement involvement on ADCC. ADCC was confirmed in the presence or absence of heat inactivation in M0 and BMDM (−) groups. In M0 (**A**) as well as BMDM (−) (**B**) groups, complement inactivation showed little effect on ADCC. *p < 0.05.

## Data Availability

All data and materials included in this study are available upon request by contact with the corresponding author.

## Abbreviations

ADCC: Antibody-dependent cellular cytotoxicity
ADCP: Antibody-dependent cellular phagocytosis
ALS: Amyotrophic lateral sclerosis
ANOVA: Analysis of variance
BMDM: Bone marrow-derived macrophage
BMSC: Bone marrow stromal cell
CLP: Clodronate-encapsulated liposome
CNS: Central nervous system
DAPI: 4ʹ,6-Diamidino-2-phenylindole
DMEM: Dulbecco's modified Eagle's medium
FBS: Fetal bovine serum
FCM: Flow cytometry
GFP: Green fluorescent protein
HAC: Human artificial chromosome
hi-MSC: Human immortalized MSC
IFN: Interferon
IL: Interleukin
LPS: Lipopolysaccharide
MCAO: Middle cerebral artery occlusion
MSC: Mesenchymal stem cell
PBS: Phosphate-buffered saline
PFA: Paraformaldehyde
PS: Penicillin–streptomycin
