# Supplementary material for: Roles of microglia/macrophage and antibody in cell sheet transplantation in the central nervous system
Source: Stem Cell Res Ther. 2022 Sep 11;13:470. doi: 10.1186/s13287-022-03168-5 (PMC9465875; doi:10.1186/s13287-022-03168-5)
Supplement: Supplementary file 1 — Additional file 1: Fig. S1. Morphological analysis of DAPI-positive cells in DCM. After FCM, HAC-MSC fraction (CD11b-negative and DAPI-positive cells) were observed. Living cells were stained with CytoRed. CytoRed-negative and DAPI-positive cells were observed (arrows). Fig. S2. CLP administration depletes microglia and macrophage population. Confocal microscopy images of empty liposomes injected (A–C) or CLP-treated (D–F) brain surface adjacent to the HAC-MSC sheet. GFP, green; Iba1, gray. Fig. S3. Complement involvement on ADCC. ADCC was confirmed in the presence or absence of heat inactivation in M0 and BMDM (−) groups. In M0 (A) as well as BMDM (−) (B) groups, complement inactivation showed little effect on ADCC. *p < 0.05. [file 13287_2022_3168_MOESM1_ESM.docx]

Fig. S1


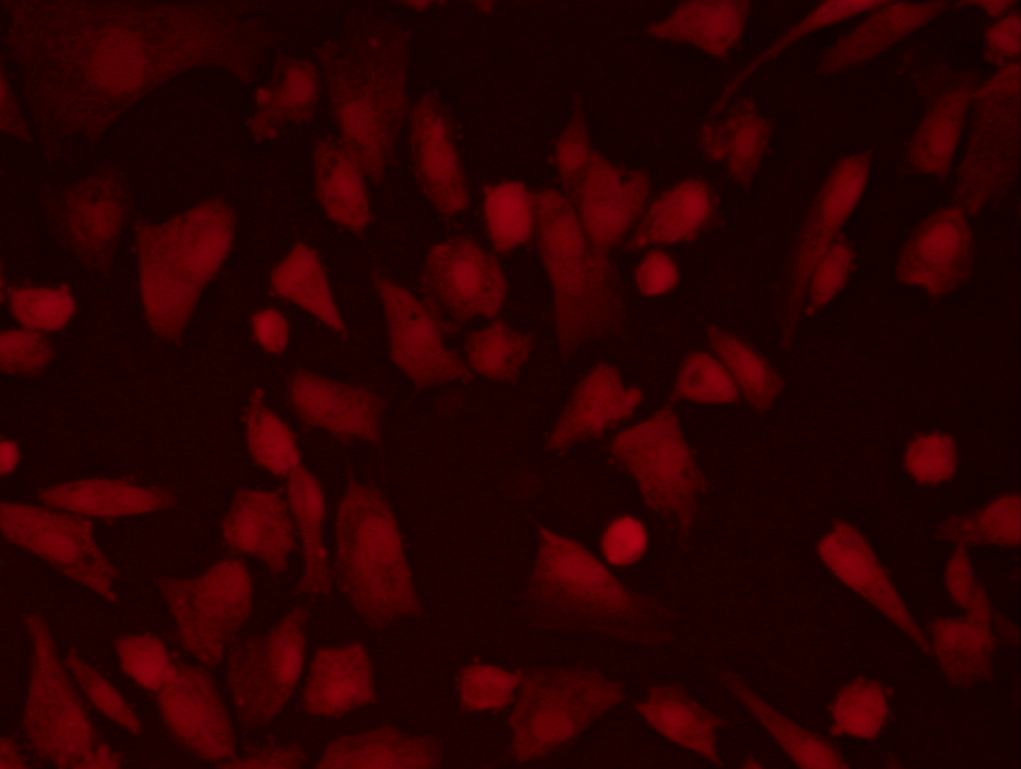

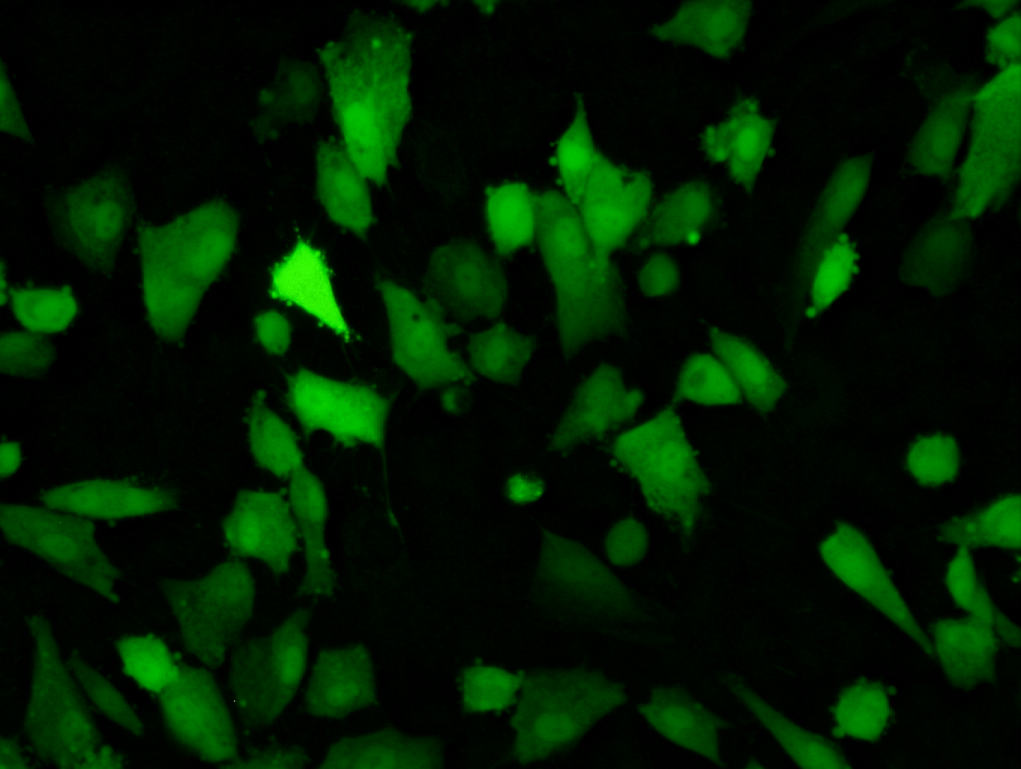

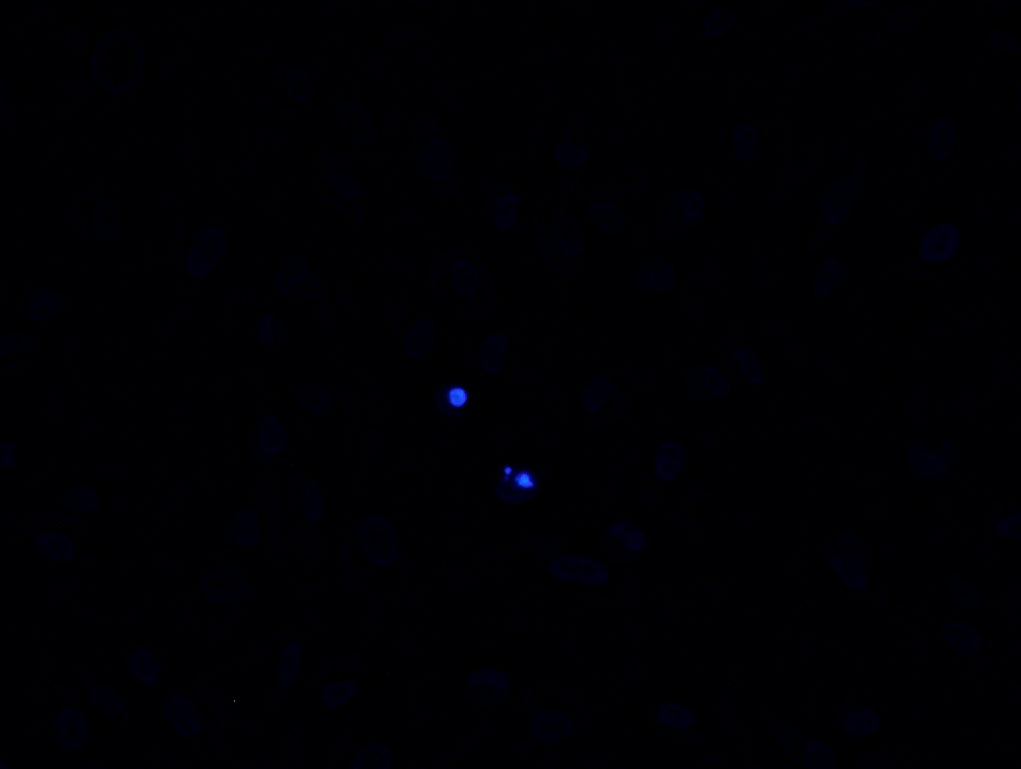

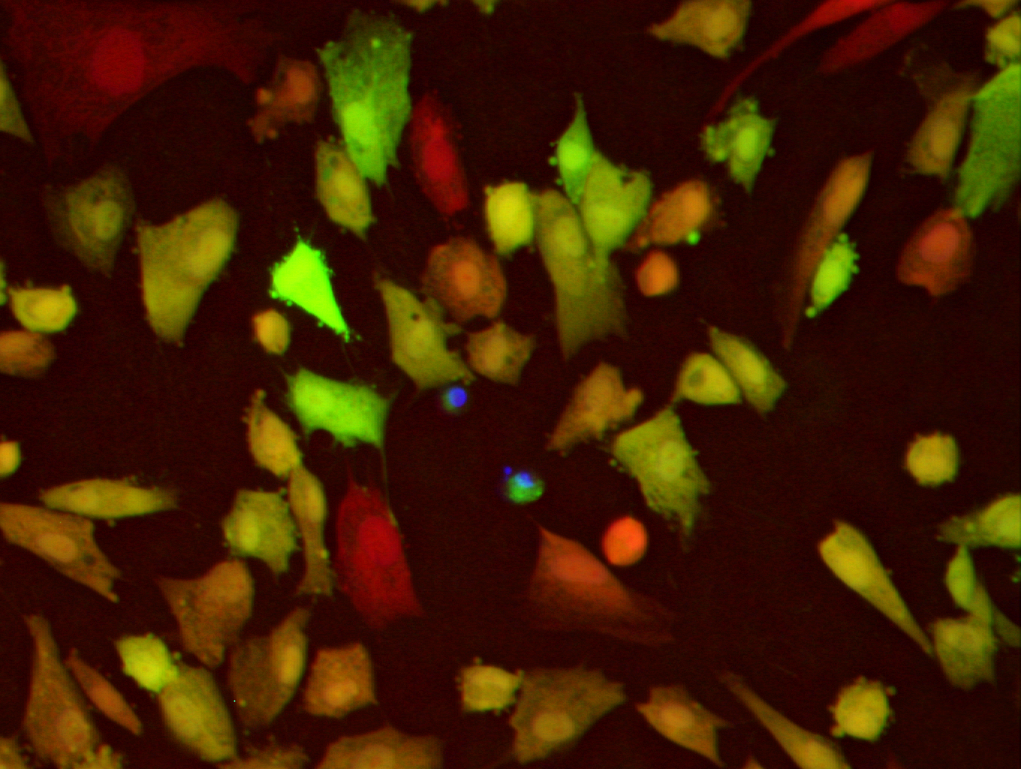


CytoRed

DAPI

GFP

Merged

20 µm

Fig. S2

Liposome

CLP


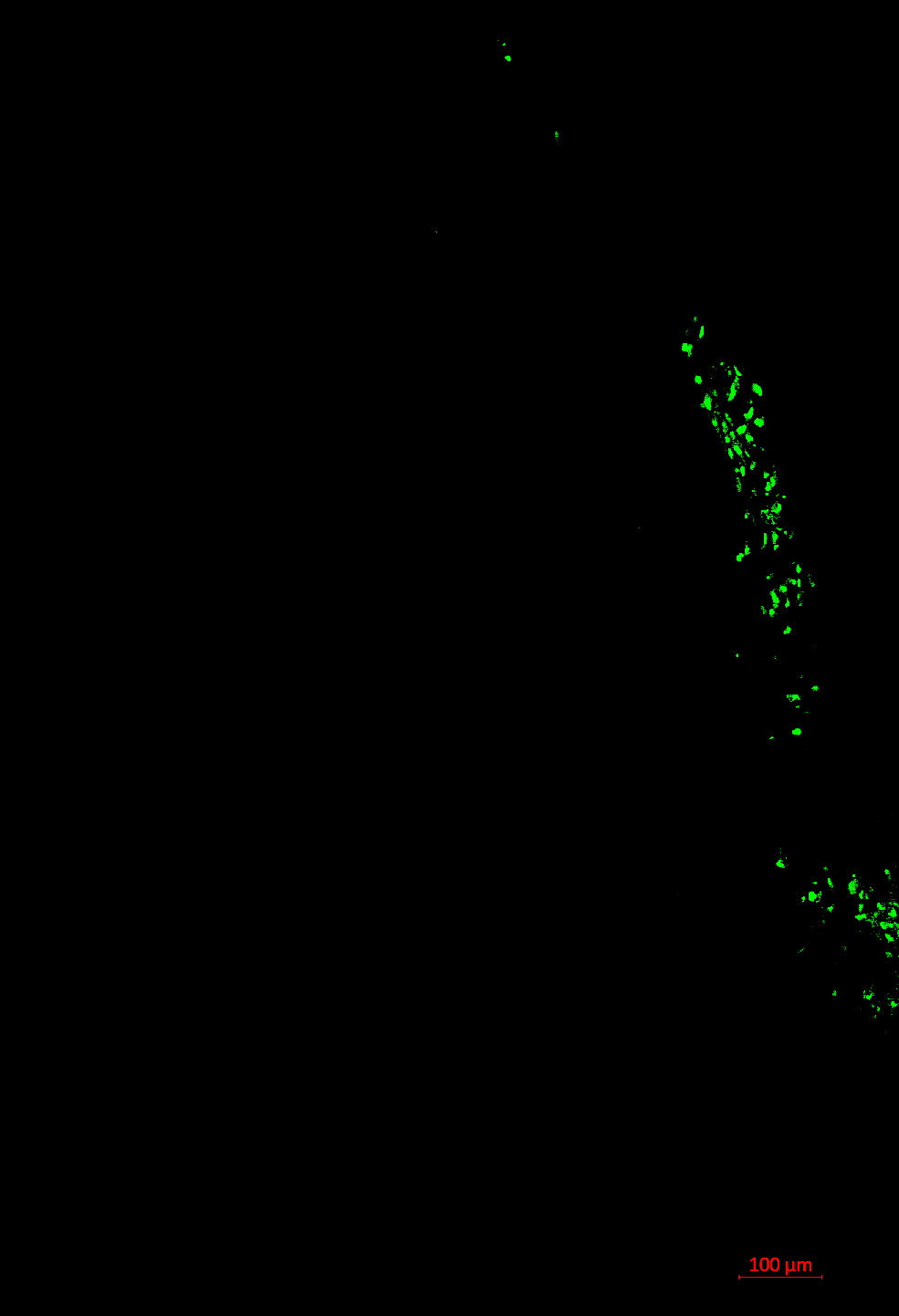

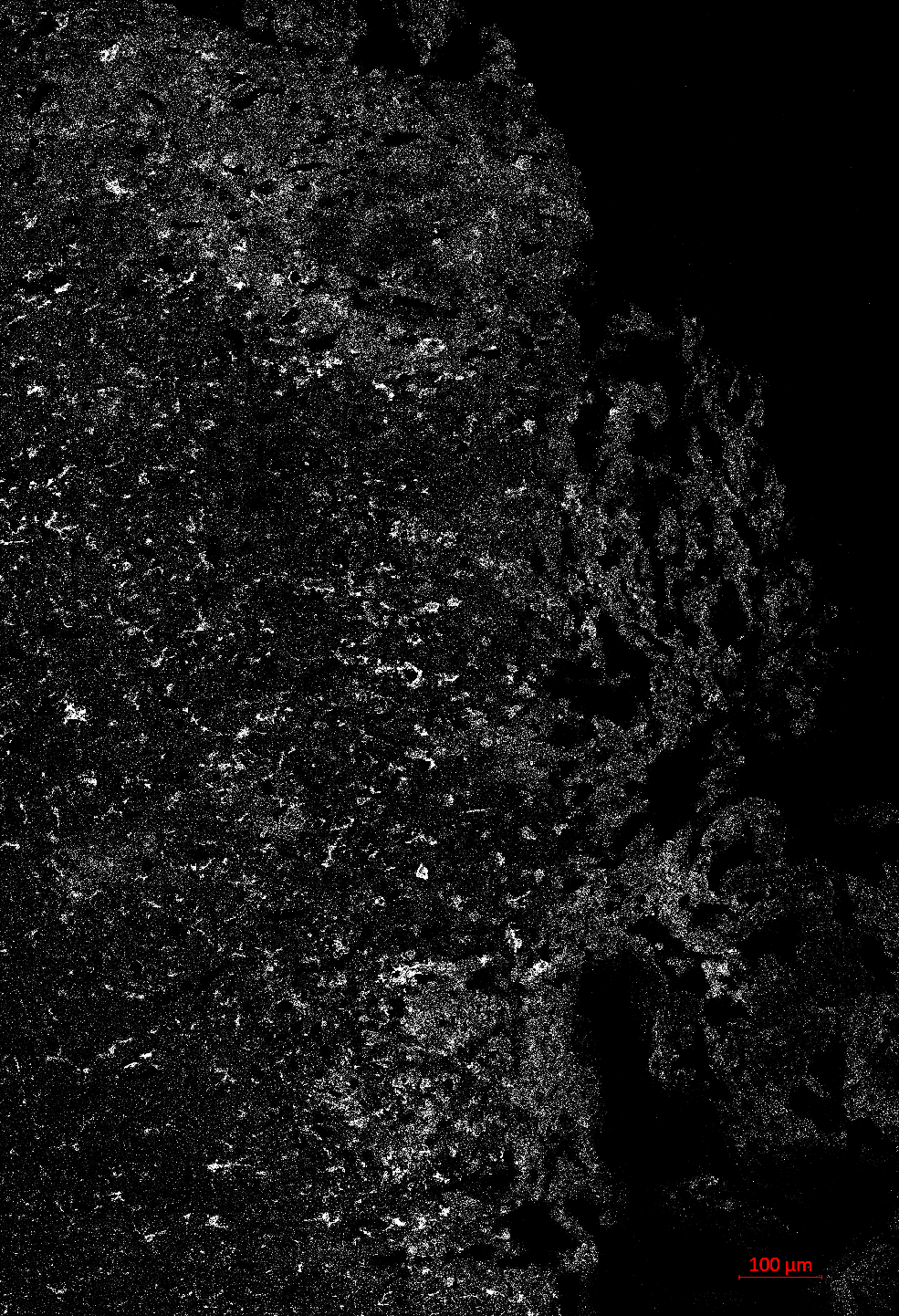

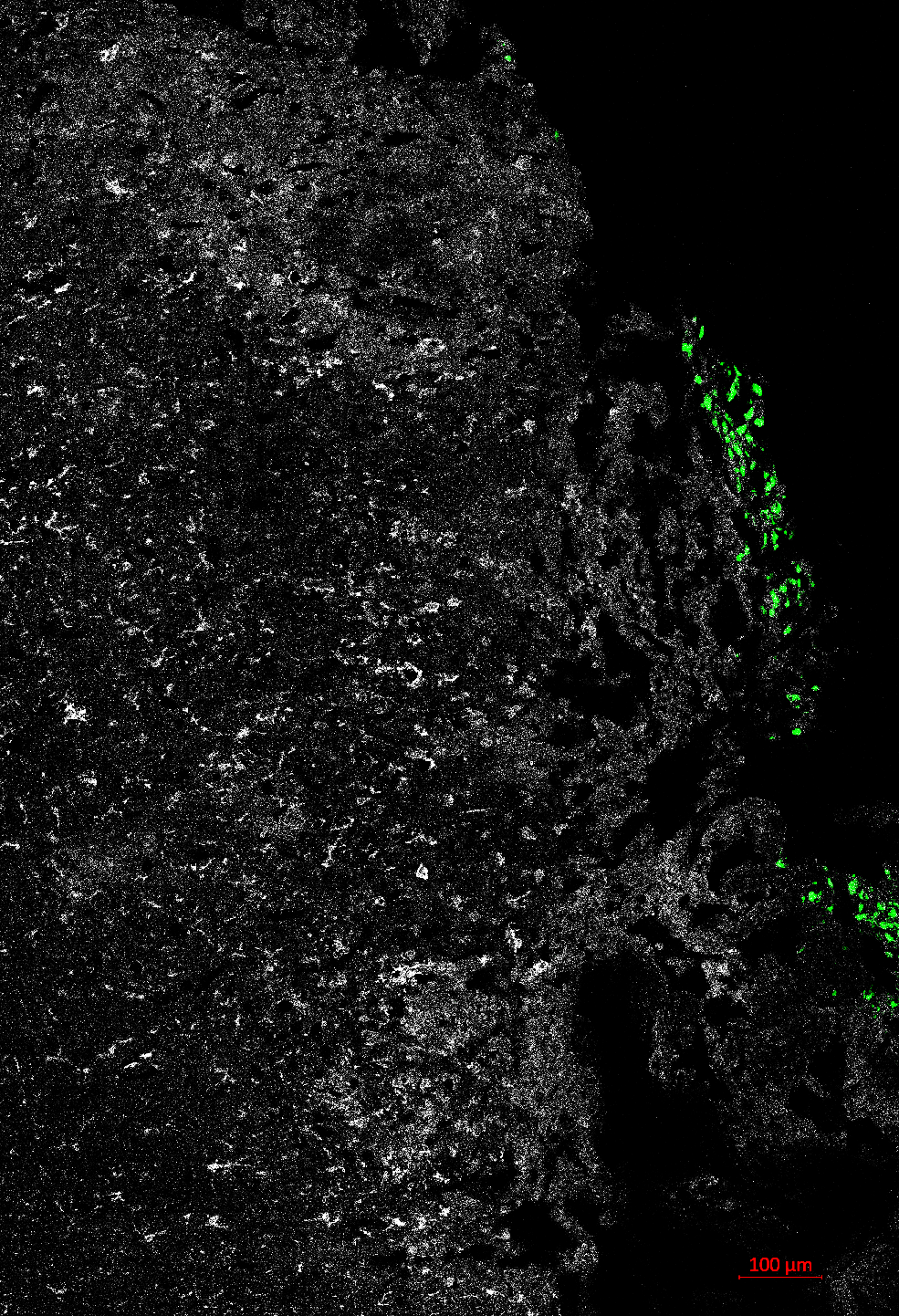

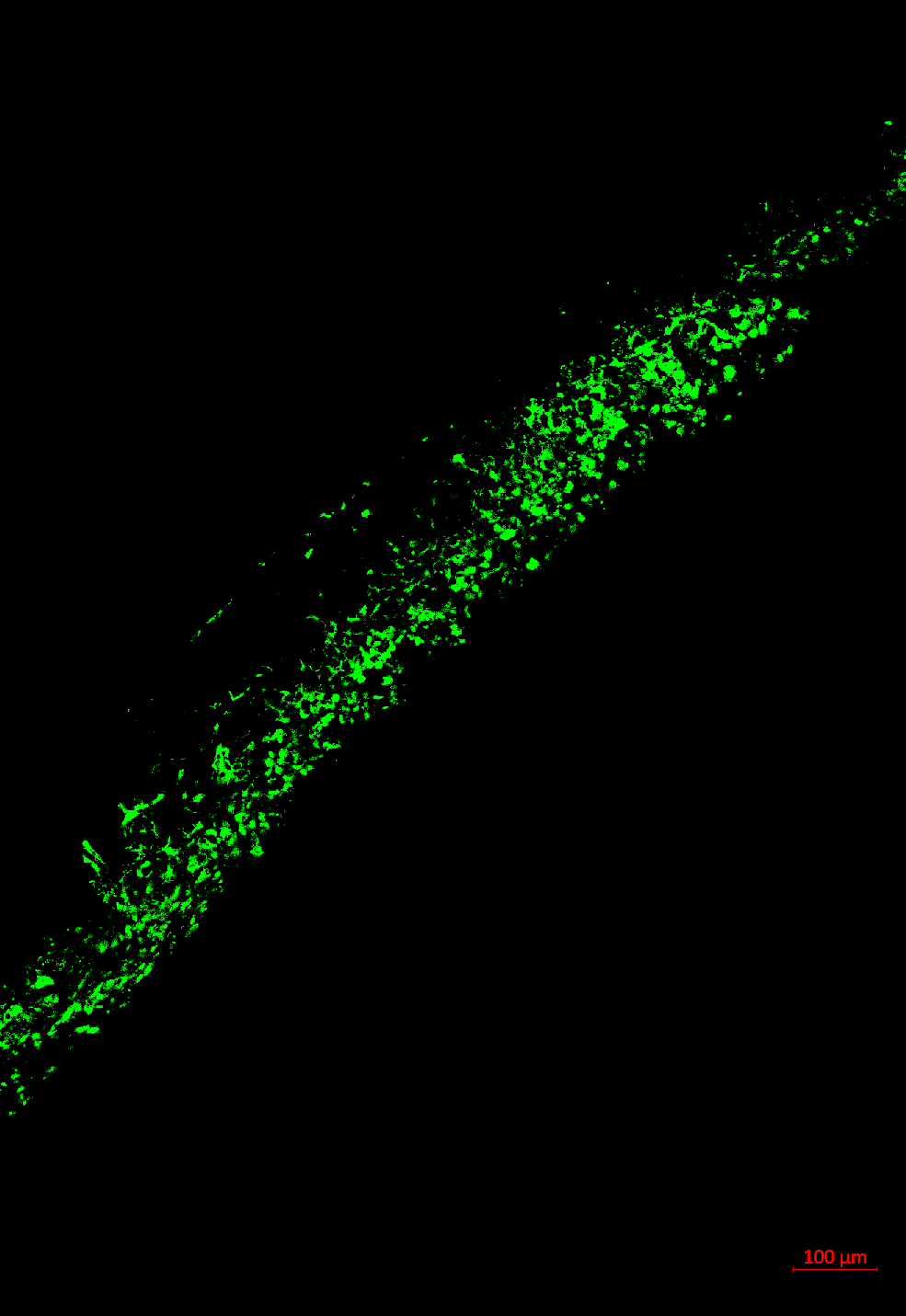

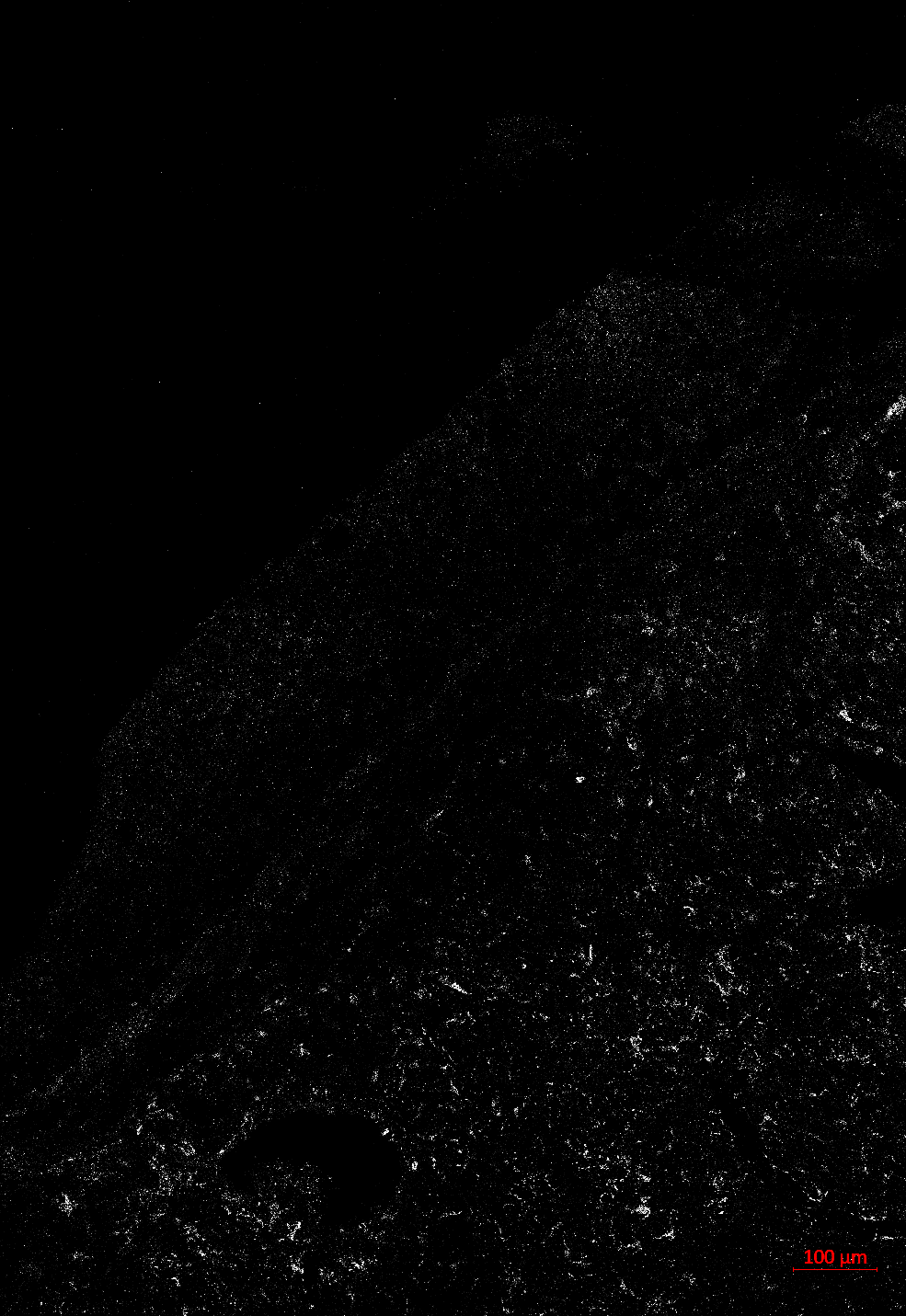

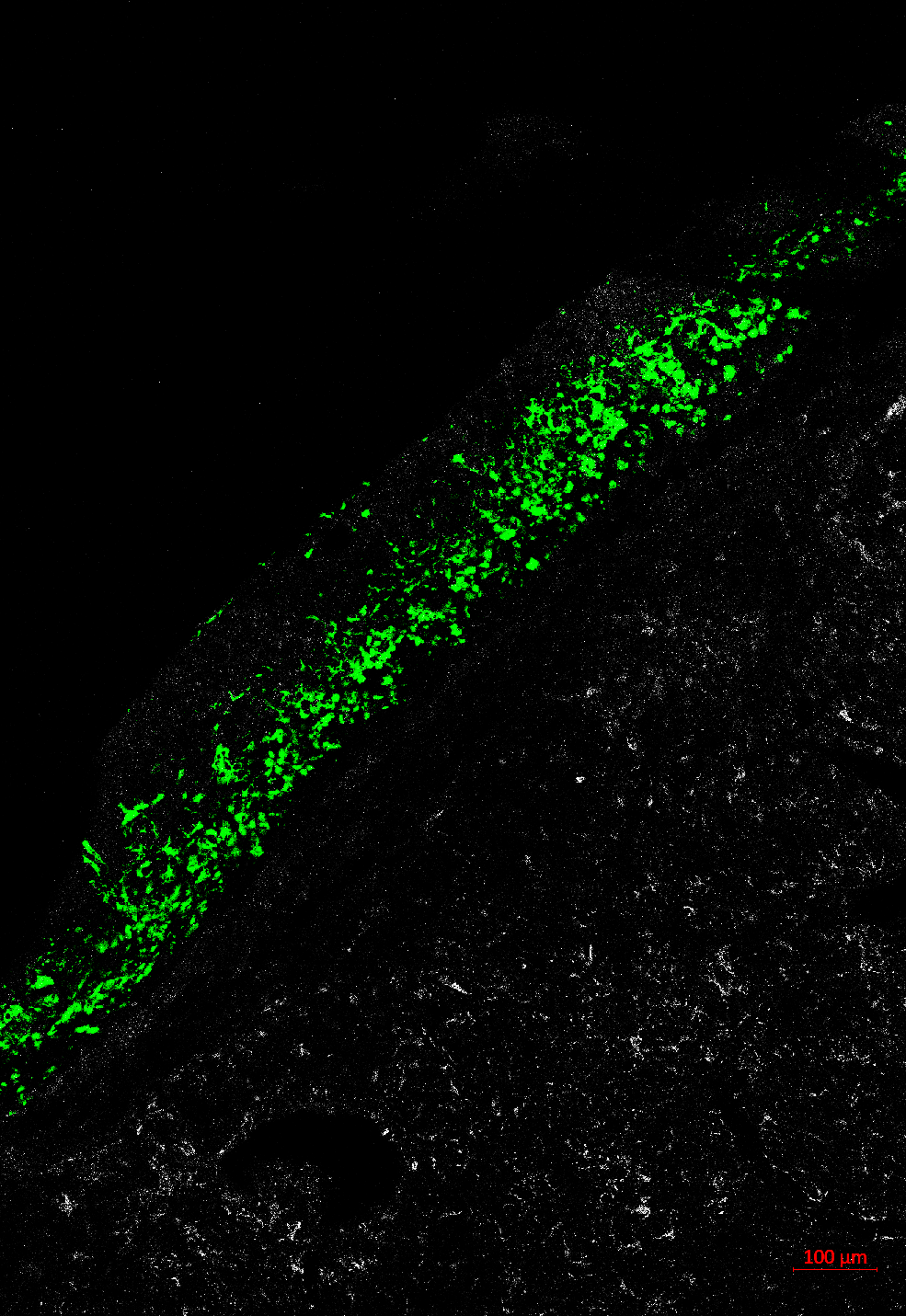


100 µm

GFP

Merged

Iba1

A

B

C

D

E

F

Fig. S3

Normal

*

*

BMDM (M0)

BMDM (-)

*

*

% Dead HAC-MSCs - fraction

(%)

(%)

Sensitized

Inactivation (-)

Sensitized

Inactivation (+)

% Dead HAC-MSCs - fraction

A

B

0

1

2

3

4

5

6

7

0

1

2

3

4

5

6

7


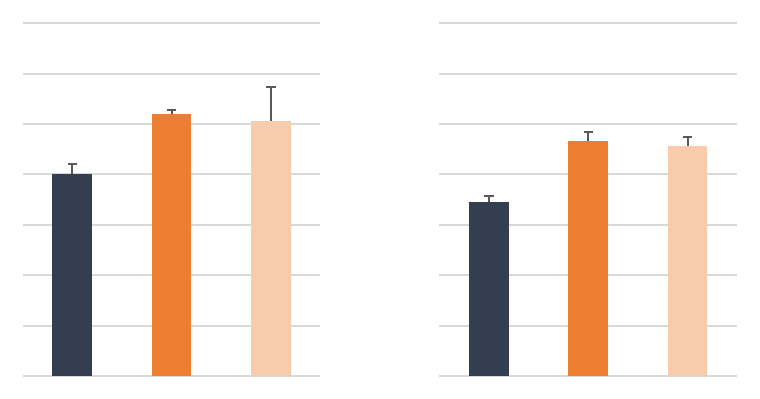


Sensitized

Inactivation (-)

Sensitized

Inactivation (+)

Normal
